# Supplementary material for: Regulating the scaling relationship for high catalytic kinetics and selectivity of the oxygen reduction reaction
Source: Nat Commun. 2022 Oct 27;13:6414. doi: 10.1038/s41467-022-34169-w (PMC9613657; doi:10.1038/s41467-022-34169-w)
Supplement: Supplementary file 1 — Supplementary Information [file 41467_2022_34169_MOESM1_ESM.pdf]

Supplementary Information

Regulating the scaling relationship for high catalytic kinetics and selectivity of the oxygen reduction reaction

Wanlin Zhou[1], Hui Su[1,2]*, Weiren Cheng[1,3], Yuanli Li[4], Jingjing Jiang[1], Meihuan Liu[1], Feifan Yu[5], Wei Wang[5], Shiqiang Wei[1], and Qinghua Liu[1]*

[1]*National Synchrotron Radiation Laboratory, University of Science and Technology of China, Hefei 230029, Anhui, P. R. China*

[2]*School of Materials Science and Engineering, Anhui University, Hefei 230601, Anhui, P. R. China*

[3]*Institute for Catalysis, Hokkaido University, Sapporo 001-0021, Japan*

[4]*Fundamental Science on Nuclear Wastes and Environmental Safety Laboratory, Southwest University of Science and Technology, Mianyang 621010, Sichuan, P. R. China.*

[5]*School of Chemistry and Chemical Engineering, Key Laboratory for Green Processing of Chemical Engineering of Xinjiang Bingtuan, Shihezi University, Shihezi 832003, China*

*E-mail: suhui@ustc.edu.cn; qhliu@ustc.edu.cn

Contents:

Supplementary Figs. 1-26;

Supplementary Tables 1-8;

Supplementary references 1-10.

Supplementary Figures and Tables.

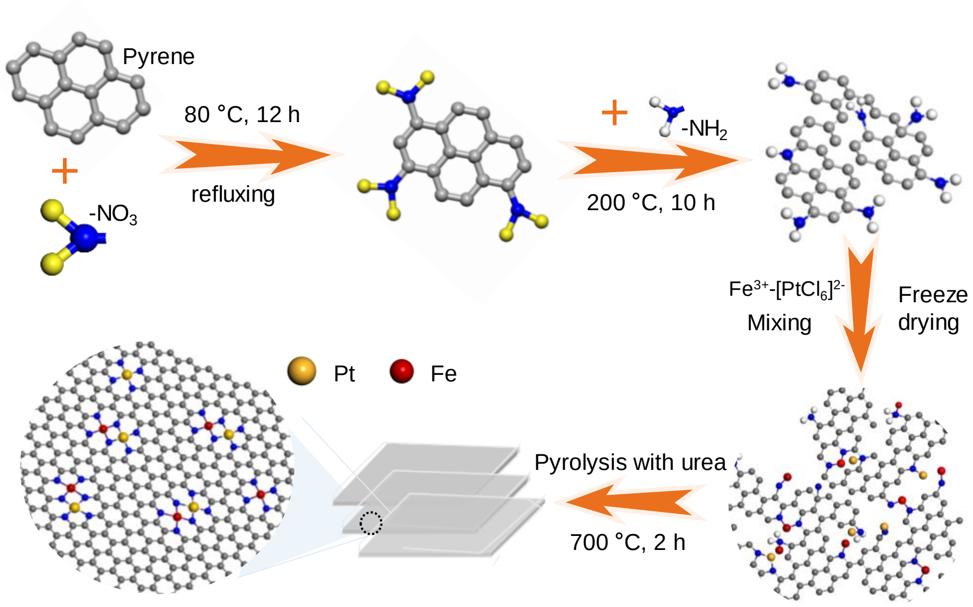

Supplementary Fig. 1 Schematic illustration for the synthetic procedure of Pt=N_2=Fe ABA.

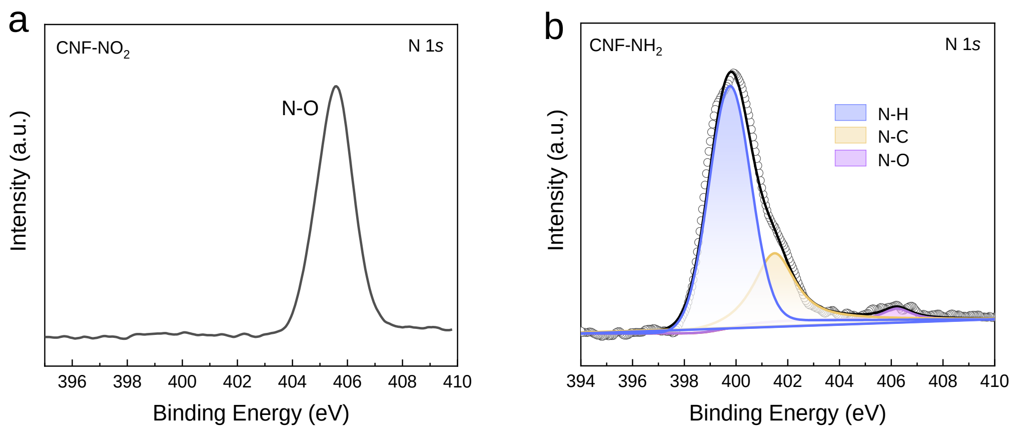

Supplementary Fig. 2 (a) N 1*s* XPS spectrum of CNF-NO_2; (b) The fitted of N 1*s* XPS spectrum of CNF-NH_2. The strong signal of N-O at 405.5 eV in (a) represent the main presence of –NO_2 in the CNF-NO_2 sample. In contrast, the deconvolution of N 1*s* XPS spectrum of CNF-NH_2 represent the main signals of N-H at 399.7 eV and N-C at 401.5 eV, respectively, and the weak signal of residual –NO_2 at 406.3 eV. It reveals that the amino group (-NH_2) almost completely replaced the nitro group (-NO_2) in the amino functionalization step to obtain the amino-functionalized carbon nanoflakes (CNF–NH_2).

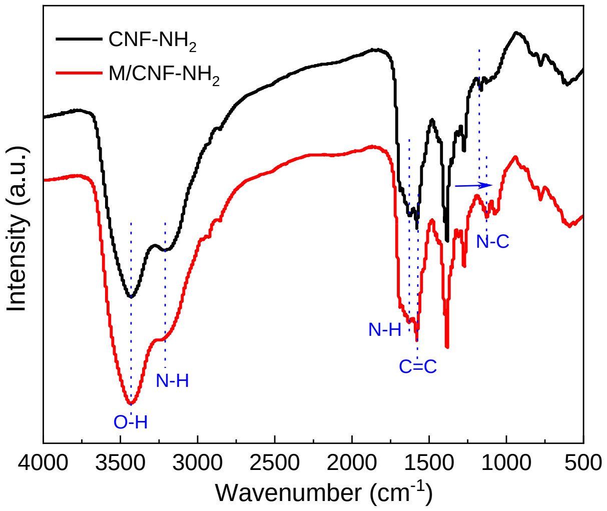

Supplementary Fig. 3 Comparative FTIR spectra of CNF-NH_2 before and after metal modification. The chemical structures are retained except for the apparent redshift and widening of the ν_{N-C} peak at ~1120 cm^{-1}, indicating the coordination of metal ions to the N atoms of the amino group.

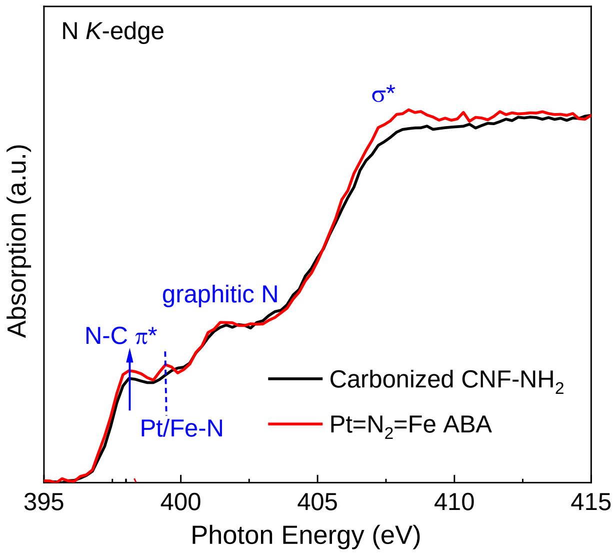

Supplementary Fig. 4 N *K*-edge XANES of Carbonized CNF-NH_2 (CNF-NH_2 annealed at 700 ℃) and Pt=N_2=Fe ABA (M/CNF–NH_2 annealed at 700 ℃). The coordination peak of metal sites(Pt/Fe) with nitrogen atoms can be clearly observed at 299.4 eV. And the enhanced N-C π* peak at 398.1 eV and broadened σ* peak at ~408.1 eV of Pt=N_2=Fe ABA attributed to the covalent coupling between metal and nitrogen, confirming the strong interaction between metal and nitrogenous matrix.

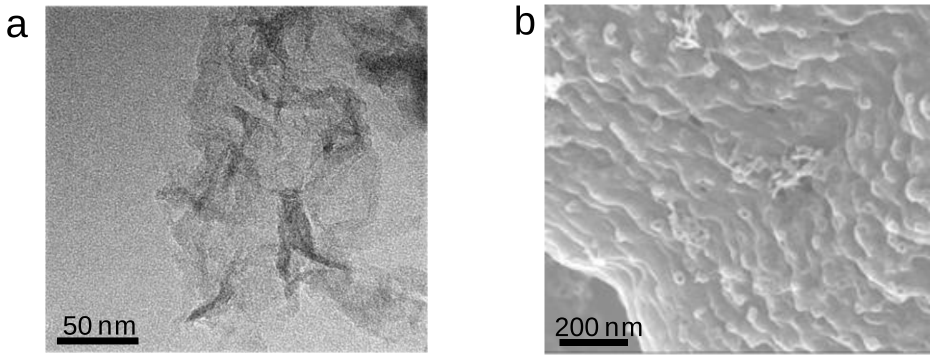

Supplementary Fig. 5 TEM (**a**) and SEM (**b**) images of Pt=N_2=Fe ABA catalyst.

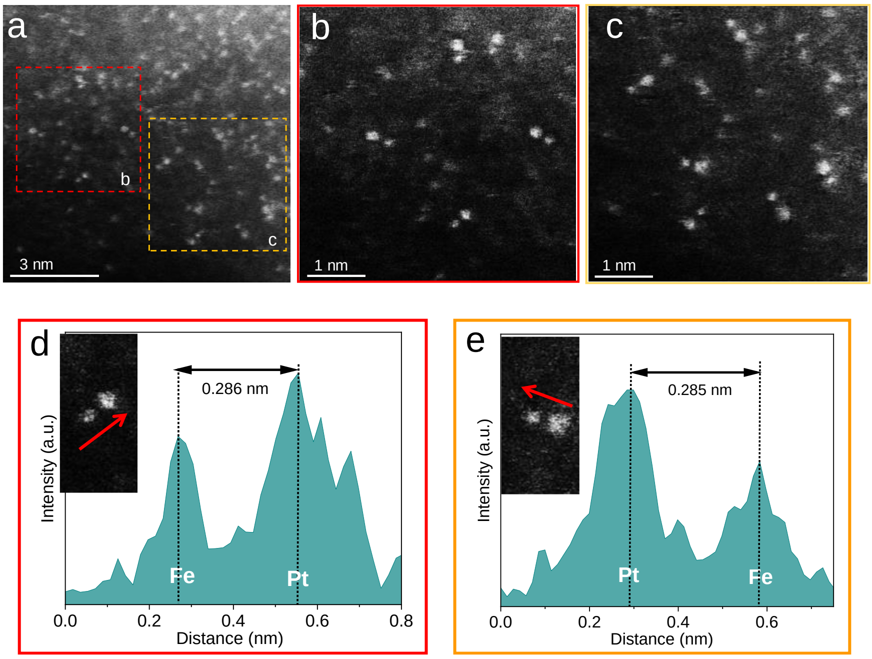

Supplementary Fig. 6 Characterizations of Pt-Fe dual-sites. **a** Cs-corrected HAADF-STEM images of Pt=N_2=Fe ABA. **b-c** Enlarged Cs-corrected HAADF-STEM images located at different positions. **d-e** The intensity profile obtained on one individual dual-sites.

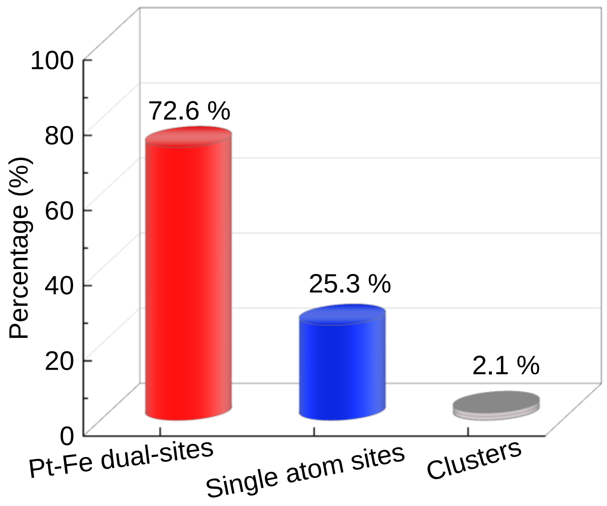

Supplementary Fig. 7 The proportional distribution of the dual-sites, single atom sites and clusters in Pt=N_2=Fe ABA sample.

.

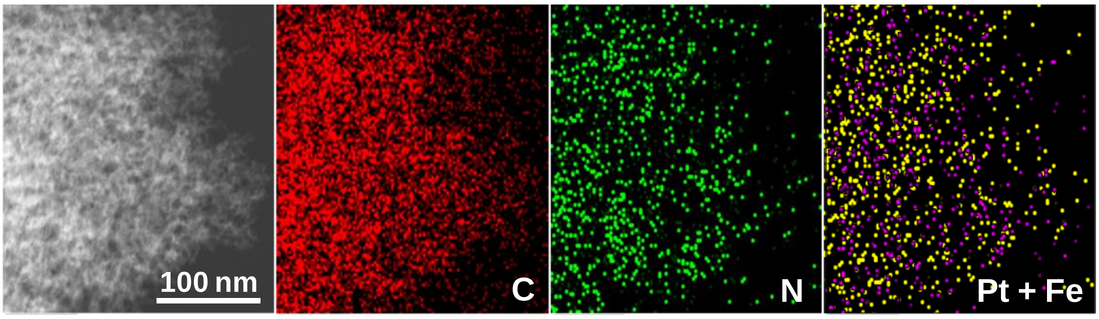

Supplementary Fig. 8 TEM-EDS mapping images for Pt=N_2=Fe ABA.

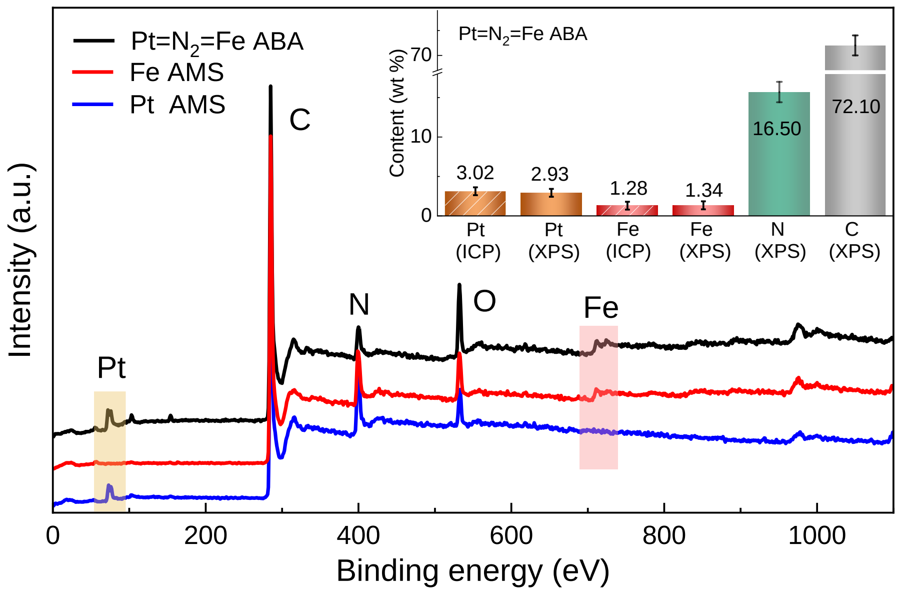

Supplementary Fig. 9 XPS survey spectra. The Fe element is absent in Pt AMS, and Pt element is absent in Fe AMS. Inset is the content display of Pt, Fe, N and C in Pt=N_2=Fe ABA measured by XPS and ICP-OES. The concentrations of Pt in Pt AMS and Fe in Fe AMS determined by XPS are about 6.65 and 1.96 wt%, which are consistent with the results of ICP-OES, respectively. The error bars were determined by the deviation of three individual measurements.

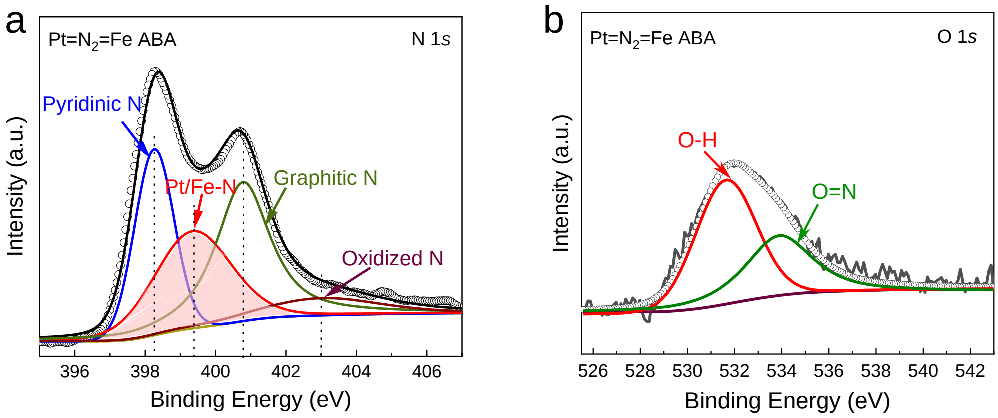

Supplementary Fig. 10 High-resolution N 1*s* and O 1*s* X-ray photoelectron spectroscopy (XPS) spectra of Pt=N_2=Fe ABA. The fitted peak of O-H bond at 531.6 eV is associated with surface-adsorbed H_2O molecules, and the fitted peak of O=N bond at 533.9 eV arises from the residual -NO_2 group that has not been completely replaced in the precursor.

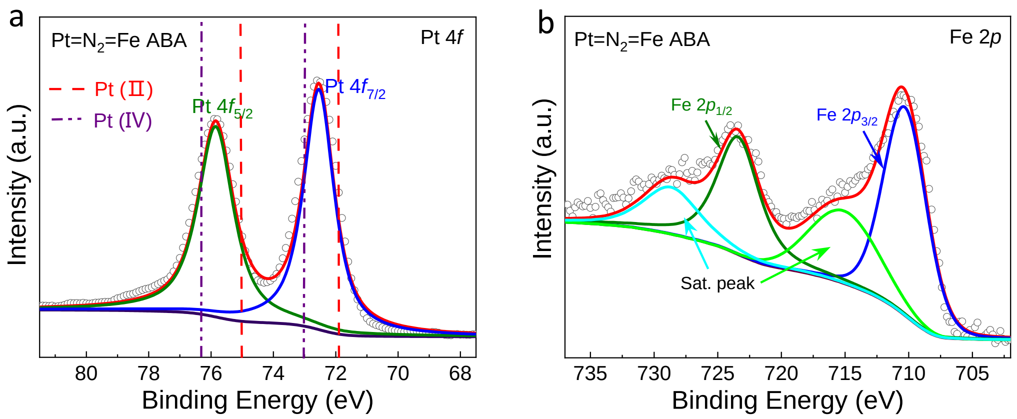

Supplementary Fig. 11 (a) Pt 4*f*, (**b**) Fe 2*p* XPS spectra of Pt=N_2=Fe ABA.

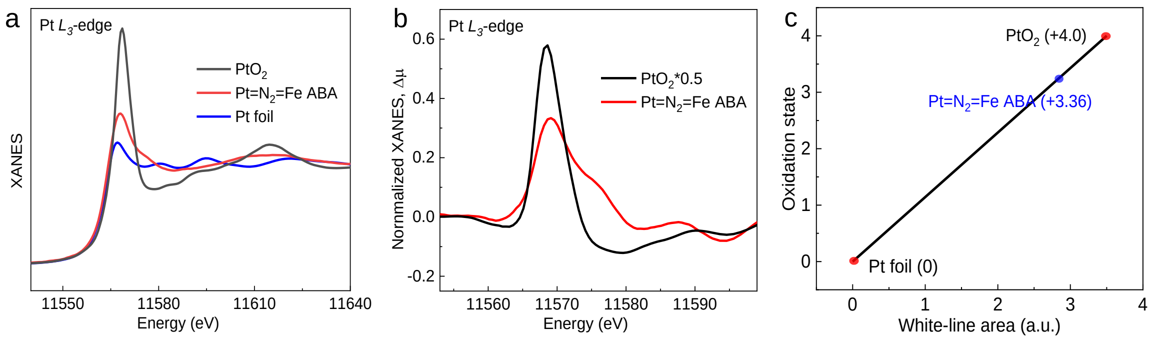

Supplementary Fig. 12 a, Pt L_3-edge XANES spectra for Pt=N_2=Fe ABA sample and the reference standards of Pt foil and PtO_2. **b,** Normalized difference spectra for Pt L_3–edge XANES using Pt foil as reference. The oxidation states are fitted through integrating the area of the white-line peak from 11560.0 to 11580.0 eV. **c,** The fitted oxidation states of Pt from ΔXANES spectra.

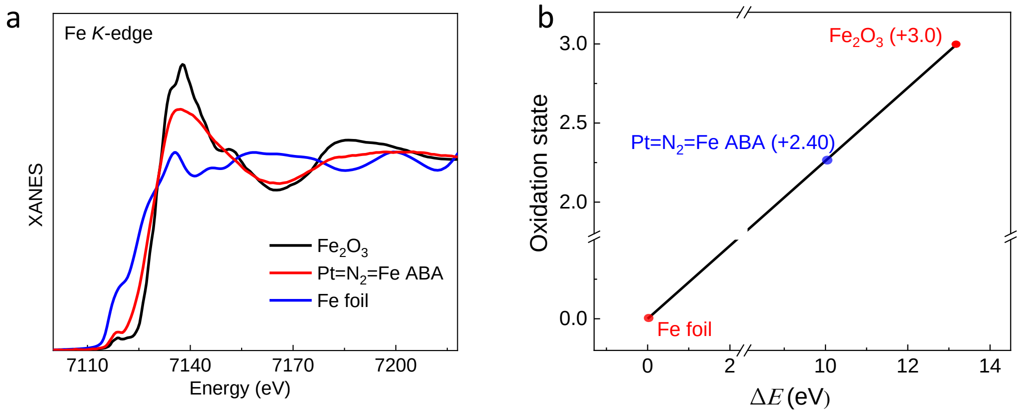

Supplementary Fig. 13 a, Fe *K*-edge XANES spectra for Pt=N_2=Fe ABA sample and the reference standards of Fe foil and Fe_2O_3. **b,** The fitted oxidation states of Fe from XANES spectra.

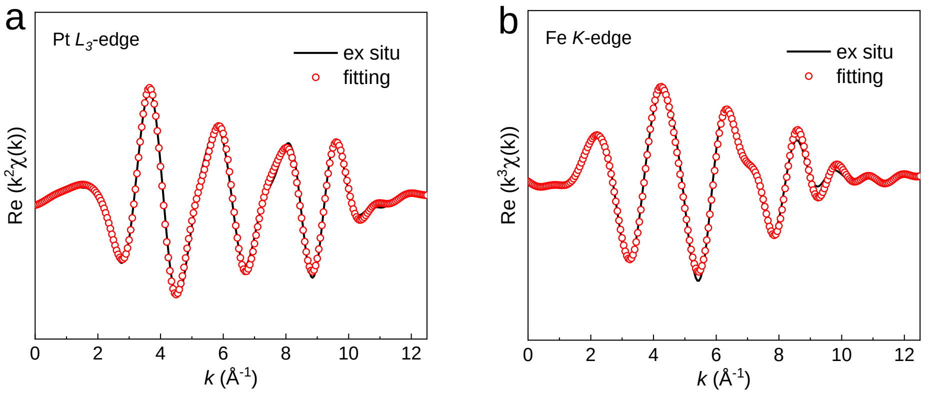

Supplementary Fig. 14 The (a) Pt L_3-edge Re($k^2\chi(k)$) and (b) Fe K-edge Re($k^3\chi(k)$) oscillation and fitting curves for Pt=N_2=Fe ABA electrocatalyst under ex situ.

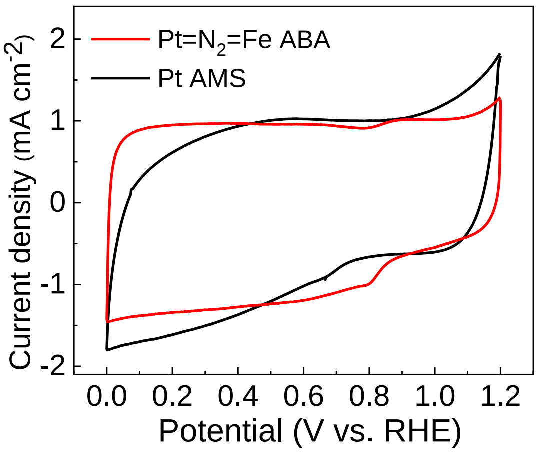

Supplementary Fig. 15 Cyclic voltammetry curves of Pt=N_2=Fe ABA and Pt AMS catalysts recorded at the potential range of 0–1.2 V vs. RHE and 50 mV s^{-1} scan rate in N_2-saturated 0.1 M KOH electrolyte.

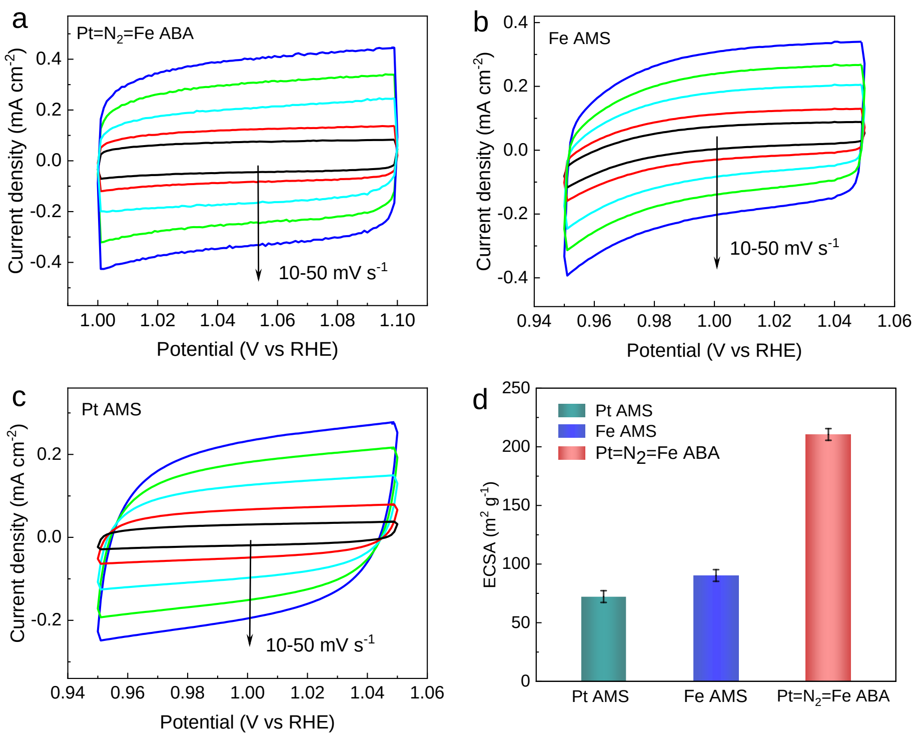

Supplementary Fig. 16 a-c, The cyclic voltammetry curves of Pt=N_2=Fe ABA catalyst and the reference samples with the scanning rates from 10 to 50 mV s^{-1}. **d,** ECSA values calculated from potential cycling. The gravimetric double layer capacitance C (F/g) can be related to the ratio of the capacitance current density *J* (mA cm^{-1}) to the scan rate range Δν (mV s^{-1}) and mass of catalyst deposited on the electrode (m, 1 g m^{-2}): C = J/Δν m. Then the ECSA (m^2 g^{-1}) can be estimated as the specific value from the gravimetric capacitance C by the equation: ECSA = C/C_s, where C_s is the double layer capacitance (F m^{-2}) of the glassy carbon electrode surface, for which the typical value of 0.4 F m^{-2} was used in KOH solution.

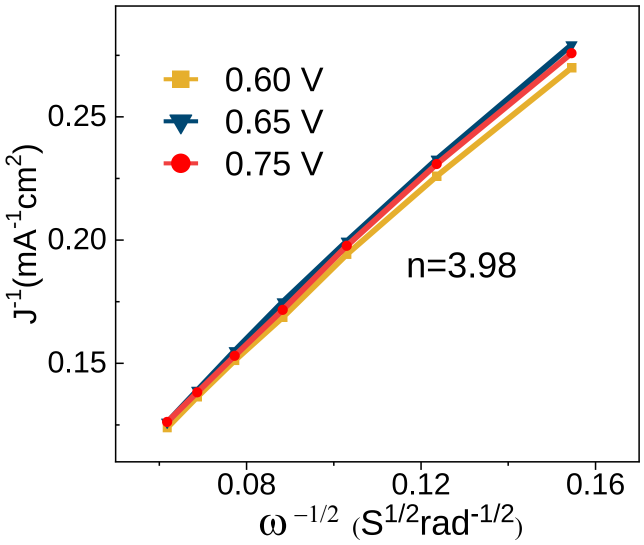

Supplementary Fig. 17 The Levich polts at different potentials derived from the polarization plots at regular rotation rates for Pt=N_2=Fe ABA.

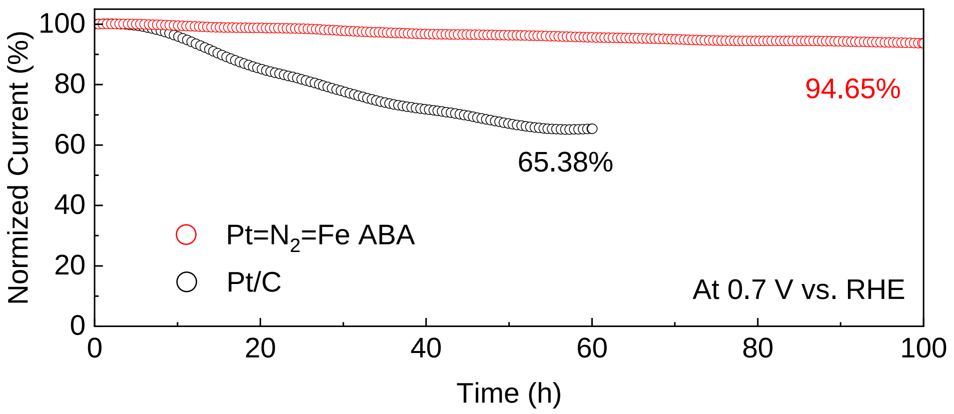

Supplementary Fig. 18 Normalized chronoamperometry curves of Pt=N_2=Fe ABA and Pt/C at constant potentials of 0.7 V vs. RHE.

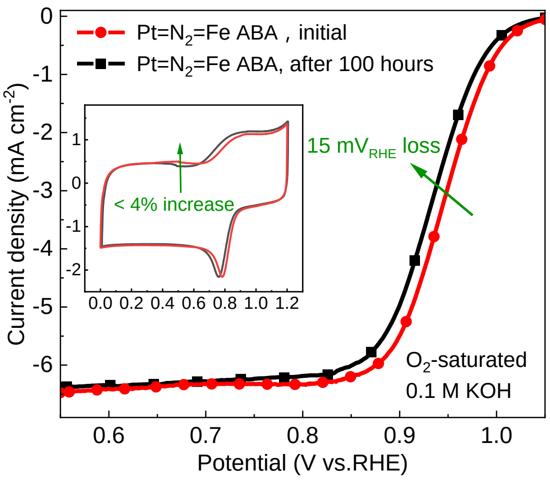

Supplementary Fig. 19 Steady-state ORR polarization plots and cyclic voltammetry curves before and after constant potential tests at 0.7 V vs. RHE for 100 hours in O_2-saturated 0.1 M KOH electrolyte.

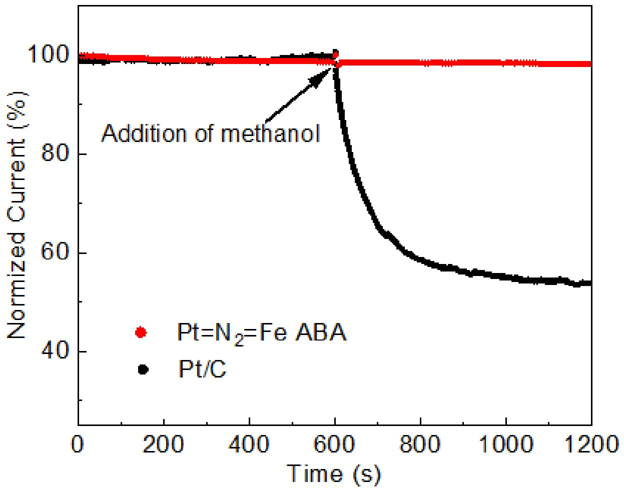

Supplementary Fig. 20 Methanol tolerance tests.

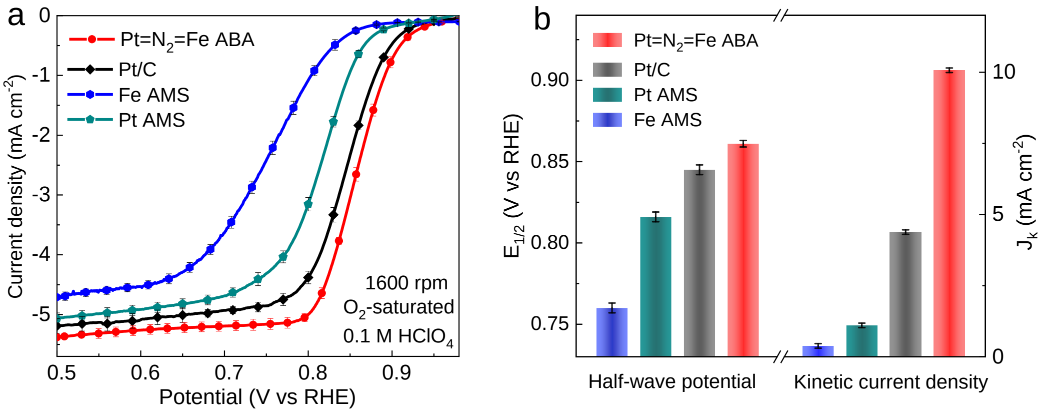

Supplementary Fig. 21 Comparison of the ORR performance between Pt=N_2=Fe ABA sample and the reference catalysts in O_2-saturated 0.1 M $HClO_4$. Error bars represent the standard deviation from at least three independent measurements. The loading of Pt=N_2=Fe ABA catalyst was 0.08 mg cm^{-2} to avoid overloading.

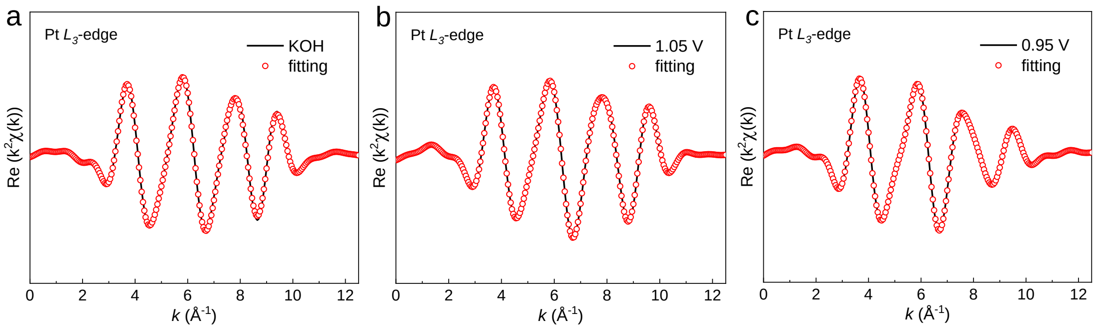

Supplementary Fig. 22 The Re($k^2\chi(k)$) oscillation and fitting curves of Pt L_3-edge for Pt=N_2=Fe ABA under (**a**) KOH solution without potential applied, (**b**) 1.05 V vs. RHE and (**c**) 0.95 V vs. RHE.

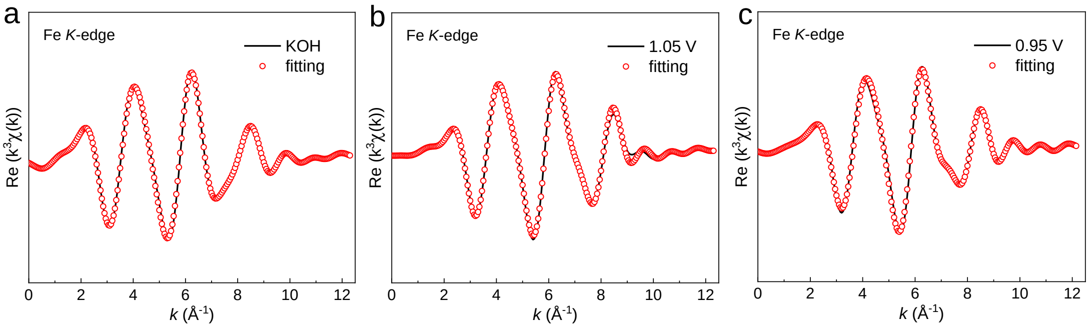

Supplementary Fig. 23 The Re($k^3\chi(k)$) oscillation and fitting curves of Fe *K*-edge for Pt=N_2=Fe ABA under (**a**) KOH solution without potential applied, (**b**) 1.05 V vs. RHE and (**c**) 0.95 V vs. RHE.

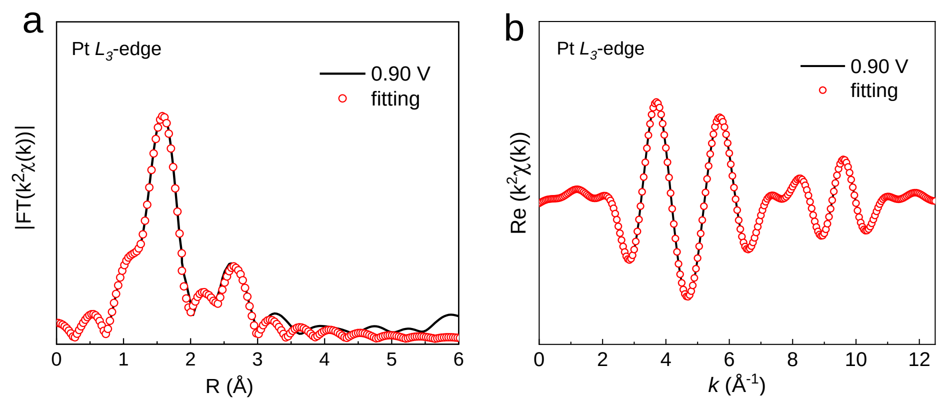

Supplementary Fig. 24 a, the fitting curve of Pt L_3-edge k^2-weighted EXAFS spectrum, and **b,** the Re($k^2\chi(k)$) oscillation and fitting curve for Pt=N_2=Fe ABA under 0.90 V vs. RHE.

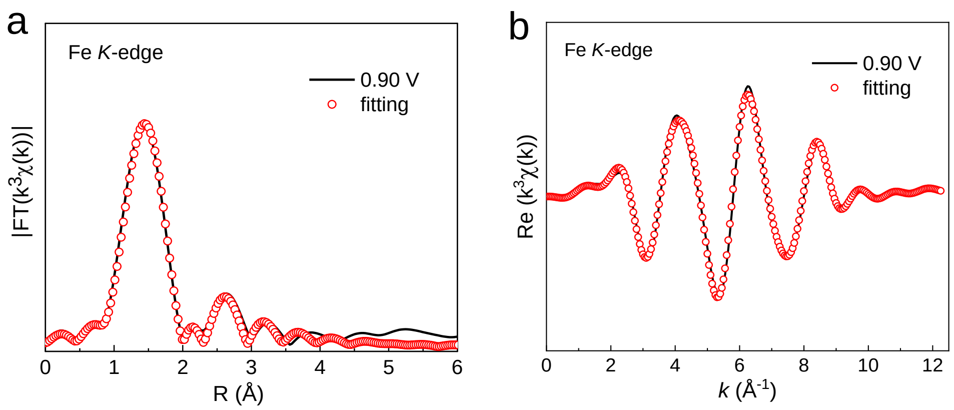

Supplementary Fig. 25 a, the fitting curve of Fe *K*-edge k^3-weighted EXAFS spectrum, and **b**, the Re($k^3\chi(k)$) oscillation and fitting curve for Pt=N_2=Fe ABA under 0.90 V vs. RHE.

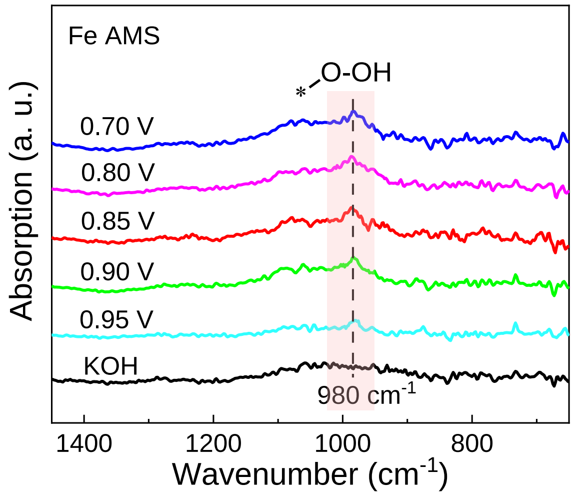

Supplementary Fig. 26 600–1500 cm^{-1} range of the in-situ SR-FTIR characterization for Fe AMS catalyst.

Supplementary Table 1. Fitting parameters of N 1s XPS spectra for Pt=N_2=Fe ABA sample.

Fitting of N 1s	Position (eV)	FWHM (eV)	Area
Pyridinic N	398.26	1.32	14640.93
Pt/Fe-N	399.35	2.58	15931.34
Graphitic N	400.78	1.82	20752.82
Oxidized N	403.01	3.60	4900.56

Supplementary Table 2. Fitting parameters of Pt 4f XPS spectra for Pt=N_2=Fe ABA sample.

Fitting of Pt 4f	Position (eV)	FWHM (eV)	Area	
Pt $4f_{5/2}$	75.85	1.25	11748.9	4 : 3
Pt $4f_{7/2}$	72.54	1.27	8823.47	

Supplementary Table 3. Fitting parameters of Pt 4f XPS spectra for Pt=N_2=Fe ABA sample.

Fitting of Fe 2p	Position (eV)	FWHM (eV)	Area	
Fe $2p_{3/2}$	710.36	2.90	12045.29	1.6 : 1
Fe $2p_{1/2}$	723.40	2.92	7601.47	
Fe $2p_{3/2}$ sat.	715.10	4.2	5806.03	
Fe $2p_{1/2}$ sat.	728.56	4.1	3504.41	

Supplementary Table 4. Structural parameters at the Pt L_3-edge for Pt=N_2=Fe ABA catalyst extracted from quantitative EXAFS curve-fittings using the ARTEMIS module of IFEFFIT.

Sample	Path	CNs	*R*(Å)	σ^2 (10^{-3} Å^2)	ΔE_0 (eV)	*R*-factor
ex situ	Pt-N	4.1±0.2	2.01±0.02	5.2±1.3	7.5	0.005
	Pt-N-Fe	1.1±0.2	2.86±0.02	7.6±1.5		
At KOH	Pt-N	4.0	1.97±0.03	6.0±1.3	5.8	0.007
	Pt-N-Fe	0.9±0.2	2.86±0.02	8.0±1.5		
At 1.05 V	Pt-N	4.0	1.99±0.02	7.2±1.5	5.5	0.009
	Pt-O	0.8±0.2	2.04±0.02	7.5±1.5		
	Pt-N-Fe	1.0±0.3	2.85±0.03	9.4±2.1		
At 0.95 V	Pt-N	4.0	1.98±0.02	7.8±1.5	5.3	0.008
	Pt-O	0.9±0.2	2.05±0.02	8.1±1.5		
	Pt-N-Fe	1.2±0.3	2.77±0.03	9.5±2.1		
At 0.90 V	Pt-N	4.0	1.95±0.02	7.5±1.5	5.0	0.008
	Pt-O	1.0±0.3	2.07±0.03	7.9±1.5		
	Pt-N-Fe	1.1±0.3	2.75±0.03	9.6±2.1		

CNs, coordination number; R, distance between absorber and backscatter atoms; σ^2, Debye–Waller factor to account for both thermal and structural disorders; ΔE_0, inner potential correction to account for the difference in the inner potential between the sample and the reference compound.; R-factor indicates the goodness of the fit. S_0^2 was determined from the Pt foil fitting as 0.90.

Supplementary Table 5. Structural parameters at the Fe *K*-edge for Pt=N_2=Fe ABA electrocatalyst extracted from quantitative EXAFS curve-fittings using the ARTEMIS module of IFEFFIT.

Sample	Path	CNs	*R*(Å)	σ^2 (10^{-3} Å^2)	ΔE_0 (eV)	*R*-factor
ex situ	Fe-N	4.2±0.2	1.95±0.02	6.3±1.0	6.3	0.004
	Fe-N-Pt	1.0±0.2	2.85±0.02	8.1±1.5		
At KOH	Fe-N	4.0	2.02±0.02	6.5±1.5	7.3	0.005
	Fe-N-Pt	0.9±0.2	2.86±0.02	9.3±2.0		
At 1.05 V	Fe-N	4.0	2.01±0.02	6.7±1.5	7.2	0.008
	Fe-N-Pt	1.2±0.3	2.84±0.03	8.0±2.0		
At 0.95 V	Fe-N	4.0	1.98±0.02	7.2±1.5	7.6	0.008
	Fe-O	1.2±0.2	2.05±0.02	7.0±1.5		
	Fe-N-Pt	1.0±0.3	2.76±0.03	9.5±2.0		
At 0.90 V	Fe-N	4.0	1.97±0.02	6.9±1.5	7.6	0.009
	Fe-O	1.1±0.2	2.08±0.02	6.8±1.5		
	Fe-N-Pt	1.1±0.3	2.74±0.03	9.8±2.0		

CNs, coordination number; R, distance between absorber and backscatter atoms; σ^2, Debye–Waller factor to account for both thermal and structural disorders; ΔE_0, inner potential correction to account for the difference in the inner potential between the sample and the reference compound.; R-factor indicates the goodness of the fit. S_0^2 was determined from the Fe foil fitting as 0.89.

Supplementary Table 6. Comparison of ORR performance of our catalysts with the reported state-of-the-art catalysts.

Catalysts	$E_{1/2}$ (V vs. RHE)	E_{onset} (V vs. RHE)	J_k (mA cm^{-2})	Tafel slope (mV dec^{-1})	Reference
Pt=N_2=Fe ABA	0.950	1.050	5.83@ 0.95 V<br>105.5@0.85 V	58	This work
PtFeNC	0.895	1.050	16.25<br>@ 0.85 V	–	1
meso/micro FeCo–Nx–CN–30	0.886	0.954	–	57	2
Fe–N/P–C–700	0.867	0.941	24.49<br>@ 0.85 V	–	3
Fe–NC SAC	0.900	0.980	–	48	4
Fe,Mn/N–C	0.928	0.979	7.04<br>@ 0.93 V	79	5
Ni–N_4/GHSs /Fe–N_4	0.830	0.930	–	55	6
Fe@Aza–PON	0.839	0.900	–	60	7
N–doped C/CNTs	0.820	0.720	8.30<br>@ 0.80 V	68.1	8
Cu/Zn–NC	0.830	0.980	–	54.8	9
Mn/Fe–HIB–MOF	0.883	0.980	–	36	10

Supplementary Table 7. Comparison of RDE mass activity of Pt=N_2=Fe ABA with several high-performance catalysts from published works.

Catalyst	Mass activity<br>A mg^{-1}_{Pt} @0.9 V vs. RHE	Reference
Pt=N_2=Fe ABA	14.1	*This work*
LP@PF-2	12.36	*Science*<br>2018, 362, 1276–1281
Mo-Pt_3Ni/C	6.98	*Science*<br>2015, 348, 1230–1234
Pt_3Ni/C nanoframe	5.7	Science<br>2014, 343, 1339–1343
PtNiCo NW	4.2	*Sci. Adv.*<br>2017, 3 1601705
sd-$Pt_{84}Ni_{12}Co_4$	7.1	*Nat. Catal.*<br>2022, 5, 513-523
PtCo-NC-3	4.16	*Research*<br>2020, 9167829
J-PtNWs/C	13.6	*Science*<br>2016, 354, 1414–1419

Supplementary Table 8. Summary of reported performance of Zn-air batteries using various electrocatalysts.

Air-cathode Catalysts	Catalyst loading (mg cm^{-2})	Peak power density (mW cm^{-2})	Specific capacity (mAh g^{-1})	Reference
Pt=N_2=Fe ABA	1.0	198.4	787.8 @10 mA cm^{-2}	This work
S–Cu–ISA/SNC	1.0	225	735 @10 mA cm^{-2}	*Nat. Comm.* **2020**,11,1-11
FeNi-DSAs /PNCH	1.0	85.03	802.18 @10 mA cm^{-2}	*Chem. Eng. J.* **2022**,437,135295
Fe–N/P–C–700	3.0	133.2	723.6 @100 mA cm^{-2}	*J. Am. Chem. Soc.* **2020**, 142, 2404
FeCu–N–HC	1.0	209.4	–	*Adv. Funct. Mater.* **2021**, 31, 2006533
Fe–SAs/NPS–HC	1.0	195	–	*Nat. Comm.* **2018**, 9,1–12.
Co_2/Fe-N@CHC	2.0	232.4	786.1 @10 mA cm^{-2}	*Adv. Mater.* **2021**, 33, 2104718
Co SAs/3D GFs	1.0	206	–	*Angew. Chem., Int. Ed.* **2020**, 132, 20645–20649
Co SA/N,S–HCS	1.5	173.1	781.1 @10 mA cm^{-2}	*Adv. Energy Mater.* **2020**, 10, 2002896

Supplementary References

1. Zhong, X. et al. Engineering Pt and Fe dual-metal single atoms anchored on nitrogen-doped carbon with high activity and durability towards oxygen reduction reaction for zinc-air battery. *Appl. Catal. B: Environ.* **286**, 119891-119898 (2021).

2. Li, S., Cheng, C., Zhao, X., Schmidt, J. & Thomas, A. Active Salt/Silica-Templated 2D Mesoporous FeCo-Nx -Carbon as Bifunctional Oxygen Electrodes for Zinc-Air Batteries. *Angew. Chem. Int. Ed.* **57**, 1856-1862 (2018).

3. Yuan, K. et al. Boosting Oxygen Reduction of Single Iron Active Sites via Geometric and Electronic Engineering: Nitrogen and Phosphorus Dual Coordination. *J. Am. Chem. Soc.* **142**, 2404-2412 (2020).

4. Zhao, L. et al. Cascade anchoring strategy for general mass production of high-loading single-atomic metal-nitrogen catalysts. *Nat. Commun.* **10**, 1278-1288 (2019).

5. Yang, G. et al. Regulating Fe-spin state by atomically dispersed Mn-N in Fe-N-C catalysts with high oxygen reduction activity. *Nat. Commun.* **12**, 1734-1733 (2021).

6. Chen, J. et al. Dual Single-Atomic Ni-N4 and Fe-N4 Sites Constructing Janus Hollow Graphene for Selective Oxygen Electrocatalysis. *Adv. Mater.* **32**, 2003134-2003144 (2020).

7. Kim, S. J. et al. Defect-Free Encapsulation of Fe(0) in 2D Fused Organic Networks as a Durable Oxygen Reduction Electrocatalyst. *J. Am. Chem. Soc.* **140**, 1737-1742 (2018).

8. Sa, Y. J. et al. Carbon nanotubes/heteroatom‐doped carbon core–sheath nanostructures as highly active, metal‐free oxygen reduction electrocatalysts for alkaline fuel cells. *Angew. Chem. Int. Ed.* **126**, 4186-4190 (2014).

9. Tong, M. et al. Operando Cooperated Catalytic Mechanism of Atomically Dispersed Cu-N4 and Zn-N4 for Promoting Oxygen Reduction Reaction. *Angew. Chem. Int. Ed.* **60**, 14005-14012 (2021).

10. Shinde, S. S. et al. Unveiling dual-linkage 3D hexaiminobenzene metal–organic frameworks towards long-lasting advanced reversible Zn–air batteries. *Energy Environ. Sci.* **12**, 727-738 (2019).
